# A novel rhamnoside derivative PL402 up-regulates matrix metalloproteinase 3/9 to promote Aβ degradation and alleviates Alzheimer’s-like pathology

**DOI:** 10.18632/aging.102637

**Published:** 2020-01-05

**Authors:** Tingting Hu, Yue Zhou, Jing Lu, Peng Xia, Yue Chen, Xin Cao, Gang Pei

**Affiliations:** 1 State Key Laboratory of Cell Biology, CAS Center for Excellence in Molecular Cell Science, Shanghai Institute of Biochemistry and Cell Biology, Chinese Academy of Sciences, University of Chinese Academy of Sciences, Shanghai 200031, China; 2 Shanghai EW Medicine Co. Ltd, Shanghai 201203, China; 3 Zhongshan Hospital Institute of Clinical Science, Fudan University, Shanghai 200032, China; 4 Shanghai Key Laboratory of Signaling and Disease Research, Collaborative Innovation Center for Brain Science, School of Life Sciences and Technology, Tongji University, Shanghai 200092, China

**Keywords:** Alzheimer’s disease, Aβ degradation, matrix metalloproteinase 3/9, learning and memory deficits, natural products derivate

## Abstract

The accumulation of amyloid-β (Aβ), considered as the major cause of Alzheimer’s disease (AD) pathogenesis, relays on the rate of its biosynthesis and degradation. Aβ degradation is a common overture to late-onset AD and targeting the impairment of Aβ degradation has gained attention in the recent years. In this study, we demonstrated a rhamnoside derivative PL402 suppressed Aβ level in cell models without changing the expression or activity of Aβ generation-related secretases. However, the levels of matrix metalloproteinase (MMP) 3 and 9, belonging to amyloid-degrading enzymes (ADEs), were up-regulated by PL402. The inhibition or the knockdown of these two enzymes abolished the effect of PL402, indicating that PL402 may reduce Aβ via MMP3/9-mediated Aβ degradation. Notably, administration of PL402 significantly attenuated Aβ pathology and cognitive defects in APP/PS1 transgenic mice with the consistent promotion of ADEs expression. Thus, our study suggests that targeting Aβ degradation could be an effective strategy against AD and the rhamnoside derivatives may have therapeutic effects.

## INTRODUCTION

Alzheimer's disease (AD), the most common form of dementia [1], is a growing global health concern with huge influence for individuals and society [1–3]. Emerging evidence suggests that amyloid-β (Aβ) is a key initiating incident in the pathogenetic process of AD [4, 5]. The accumulation of Aβ in the form of toxic oligomers leads to the formation of neuritic plaques which can cause neuronal loss, synaptic impairment and memory deficits, ultimately resulting to neurodegeneration and AD [6–9]. The level of Aβ dependents on the rate of its biosynthesis and degradation [10, 11]. The Aβ peptide is sequentially cleaved from amyloid precursor protein (APP) by two proteinases, β-secretase (mainly BACE1) and γ-secretase complex. The cleavage of APP by BACE1 generates an APP soluble fragment (sAPPβ) and a membrane-bound c-terminal fragment, which is further cleaved by a γ-secretase to produce the multiple Aβ peptides [12–15]. The Aβ peptides can be further metabolized by amyloid-degrading enzymes (ADEs) [11, 16] which include, insulin-degrading enzyme (IDE), neprilysin (NEP) and its homologue endothelin-converting enzyme (ECE), angiotensin converting enzyme (ACE) and matrix metalloproteinases (MMPs) [16–18]. Accumulating evidence has indicated that impairment of Aβ clearance is a common overture to late-onset AD [19, 20] and several *in vivo* studies demonstrated that activation of ADEs prevented Aβ accumulation and AD pathology, suggesting that these ADEs could serve as the promising therapeutic targets for the treatment of AD [18, 21–23].

Natural products and their derivatives such as glycosides are emerging drug candidates for AD therapy owing to their diverse biological functions under pathological circumstances [20, 24–26]. Among various pharmacological properties, the glycosides exhibit anti-oxidative and anti-inflammatory activities in diabetes, cardiovascular disease, and AD [19, 27]. Rhamnoside, one of the glycosides widely existing in plants, vegetables and fruits, is reported to exert anti-aging effects. We previously reported a rhamnoside derivative named PL201A could ameliorate cognitive impairments and enhance the neural progenitor cells (NPC) proliferation and neurogenesis in APP/PS1 mice [28] while whether it could influence Aβ pathology is unclear. Here we further explored the effect of PL201A and another analogue of rhamnose, PL402, on Aβ pathology and its underlying mechanism.

## RESULTS

### PL402 reduces Aβ level *in vitro*

**Figure 1:**
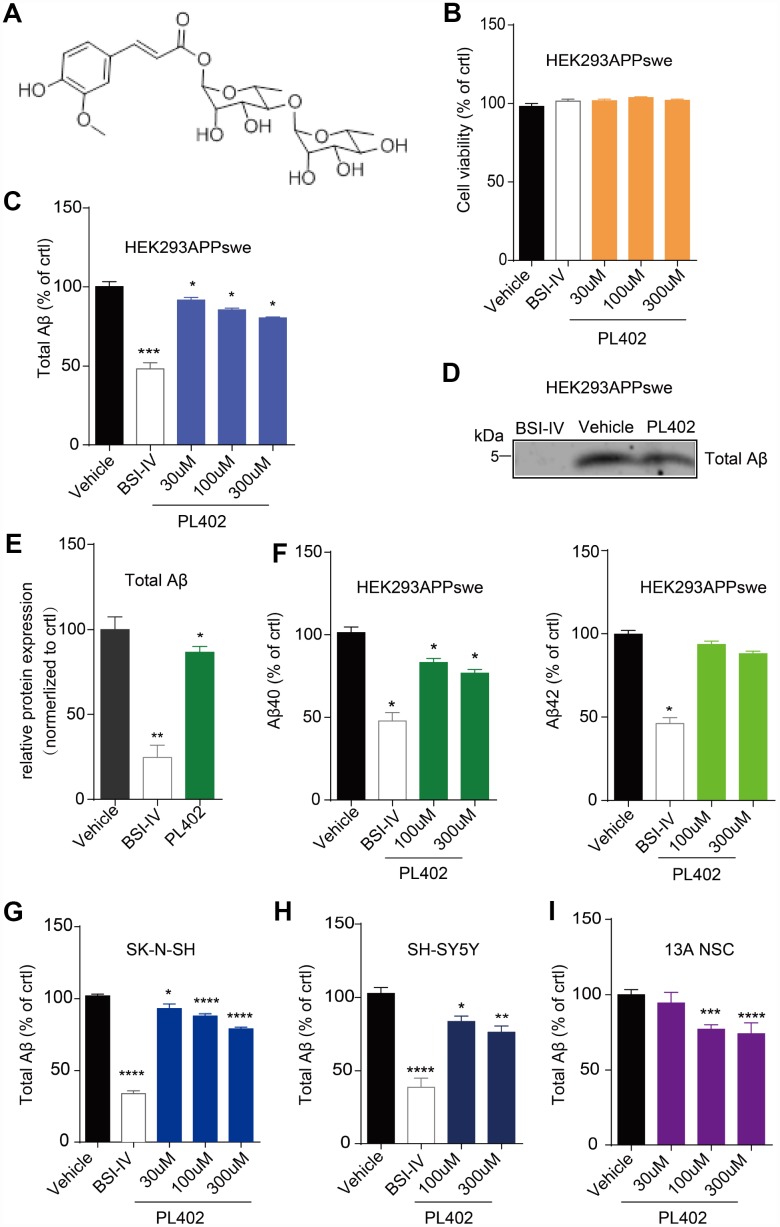
**PL402 reduces Aβ level *in vitro*.** (**A**) The structure of PL402. (**B**) The cell viability of HEK293/APPswe cells in response to vehicle (0.1% DMSO), 0.1μM BACE inhibitor IV (BSI-IV), or the PL402 at 30μM, 100μM or 300μM for 24h measured by CellTiter-Glo Assay. N=3. (**C**) The total Aβ level in the culture medium of HEK293/APPswe treated with vehicle (0.1% DMSO), 0.1μM BSI-IV, or the PL402 at 30μM, 100μM or 300μM for 24h measured by ELISA. N=5. (**D**–**E**) Representative image of a western blot showing the expression of total Aβ in HEK293/APPswe (**D**) and its quantification normalized to control. The 1μM of BSI-IV and 10μM of Forskolin were used as the positive controls. N=3 (**E**). (**F**) The level of Aβ40 or Aβ42 in the medium of HEK293APPswe examined by ELISA after treatment with vehicle (0.1% DMSO), 0.1μM BSI-IV, or PL402 at 100μM, 300μM for 24 h. N=3. (**G**–**H**) The levels of total Aβ produced by SK-N-SH (**G**) (N=9) and SH-SY5Y (**H**) (N=6) cells by ELISA in response to vehicle (0.1% DMSO), 0.1μM BSI-IV, and the PL402 at 30μM, 100μM or 300μM for 24 h. (**I**) The total Aβ level in the medium of human neural stem cells (13A NSCs) measured by ELISA after treatment with vehicle (0.1% DMSO), 0.1μM BSI-IV, or the PL402 at 30μM, 100μM or 300μM for 24 hours. N=4. The Data are presented as mean ± SEM, n >3 independent experiments, *p < 0.05, **p < 0.01, ***p< 0.001 and ****p< 0.0001 compared to the control of each group, analyzed by one-way ANOVA followed by Bonferroni test.

To identify whether PL402 (the structure shown in Figure 1A) could reduce Aβ level *in vitro*, we treated the HEK293/APPswe cells and two neuronal cell lines (SK-N-SH and SH-SY-5Y) with indicated concentrations of PL402 for 24 h. ELISA assay was performed to estimate the total Aβ level in the culture medium. Cell viability after treatments were examined using Cell Titer-Glo assay. Data showed that cell viability of two cell lines (HEK293/APPswe and SK-N-SH) was not decreased by PL402 treatment (Figure 1B, Supplementary Figure 1A). In HEK293/APPswe cells, PL402 significantly reduced the total Aβ level in a dose-dependent manner compared to the vehicle (Figure 1C). A BACE1 inhibitor (BSI-IV, 0.1 μΜ) was used as a positive control. Furthermore, we used the IP-western blot analysis to verify this phenomenon and the result confirmed that the PL402 reduced the Aβ level in culture medium (Figure 1D, 1E). In addition, both Aβ40 and Aβ42 levels were decreased by PL402 in HEK293/APPswe cells (Figure 1F). We then validated the data in SK-N-SH and SH-SY5Y, two human neuronal cell lines, and found that PL402 dose-dependently reduced the quantity of total Aβ in the cultured medium (Figure 1G, 1H). The effect of PL402 on Aβ was also detected in human neural stem cells (13A NSC) and the PL402 presented a consistent effect (Figure 1I). PL201A, an analogue of PL402, was recently reported to have anti-AD effects [28]. Here we detected the effect of PL201A on Aβ levels in HEK293/APPswe cells and SK-N-SH cells. The results showed PL201A significantly reduced the Aβ levels without affecting the cell viability (Supplementary Figure 1B–1D). All the results suggest that the designed rhamnoside derivatives, PL402 and PL201A, can reduce the Aβ level *in vitro*.

### PL402 reduces Aβ level without affecting the α/β/γ-secretase activity or altering APP processing

**Figure 2:**
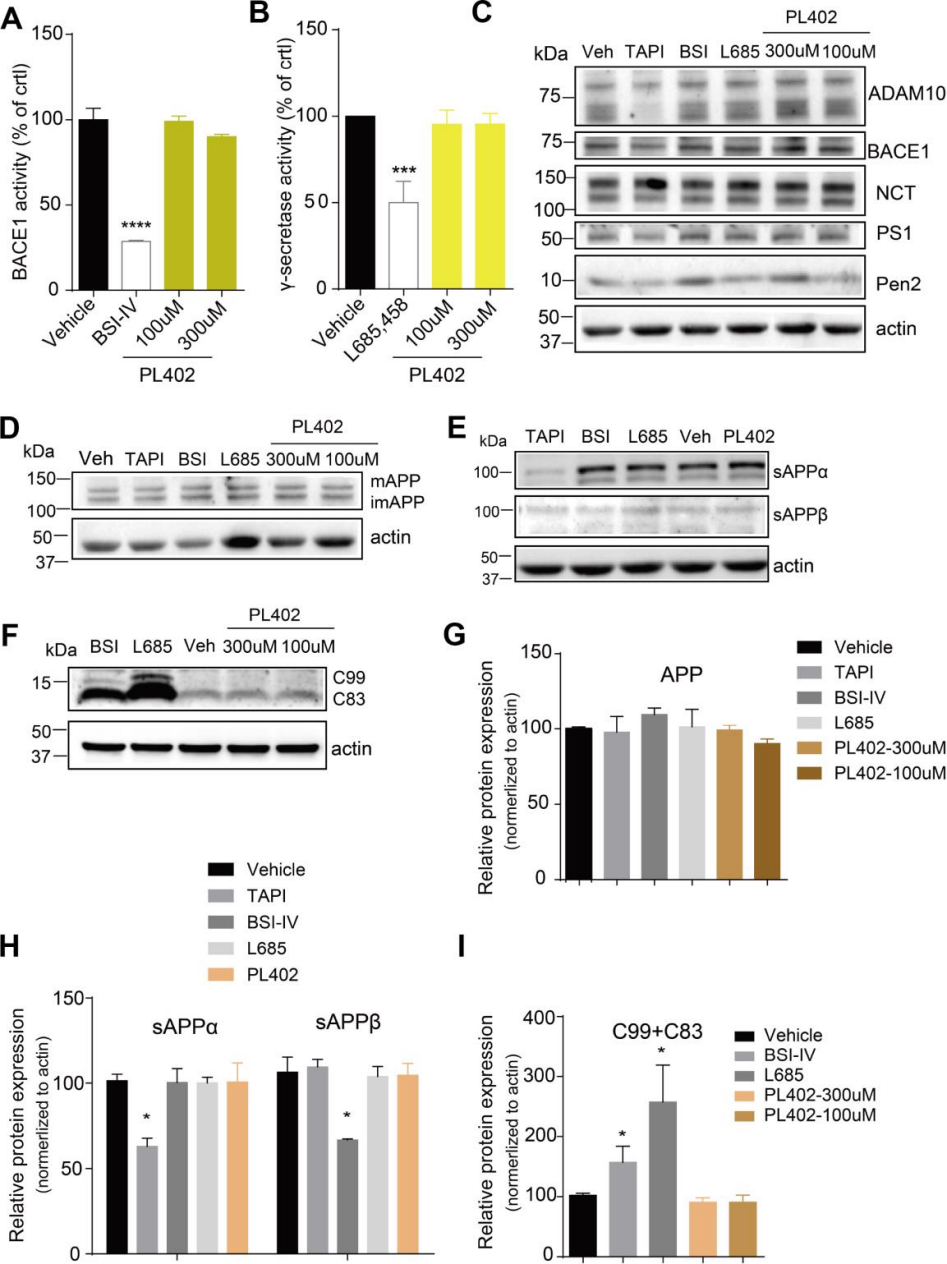
**PL402 reduces Aβ level without affecting the α/β/γ-secretase activity or altering APP processing.** (**A**, **B**) The measurements of BACE1 (**A**) and γ-secretase (**B**) activity by ELISA-based secretase assays after treatment with vehicle (0.1% DMSO), 10μM BSI IV(A), 10μM γ-secretase inhibitor L685,458 or the PL402 at 100μM, 300μM. N=3. (**C**) Representative image of a western blot showing the expression of α-secretase (ADAM10), BACE1 and γ-secretase complex (NCT, PS1, Pen2) after treatment with vehicle (0.1% DMSO), 100μM TAPI-1, 10μM BSI IV, 10μM L685,458, or 100μM and 300μM PL402 for 24 hours (**C**). Actin was used as a loading control. The statistical analysis of (**C**) was presented in Supplementary Figure 2A. N=3 (**D**–**I**) Representative image of a western blot showing the levels of mAPP, imAPP, sAPPα, sAPPβ, C99 and C83 after treatment with vehicle (0.1% DMSO), 100μM TAPI-1, 10μM BSI IV, 10μM L685,458, or 100μM and 300μM PL402 for 24 hours (**D**–**F**). N=3. (**G**–**I**) The quantification analysis of (**D**–**F**). The Data are presented as mean ± SEM, n = 3 independent experiments, *p < 0.05, ***p< 0.001 and ****p< 0.0001, analyzed by one-way or two-way ANOVA followed by Bonferroni test.

The Aβ peptide is produced by sequential cleavage of APP by BACE1 and γ-secretase [29, 30]. So, we used the ELISA-based secretase activity assays to test whether the PL402 directly influenced the two secretases activity in SK-N-SH cells. However, the PL402 showed no obvious effect on either secretase (Figure 2A, 2B), while BSI-IV significantly inhibited BACE1 activity (Figure 2A) and γ-secretase inhibitor (L685,458) distinctly inhibited γ-secretase activity (Figure 2B). And then we detected the protein level of α-secretase (ADAM10), BACE1 or γ-secretase complex (NCT, PS1-FL, Pen2) after treatment with PL402 (100 μM and 300 μM) using western blot analysis. As shown in Figure 2C and Supplementary Figure 2A, all the expression levels of above-mentioned proteins were unchanged. The results suggested that the PL402 reduces Aβ level without affecting the α-, β-, and γ-secretase activity. Previous studies showed that interfering with APP metabolism could modify Aβ level [30–32]. Thus, we observed the APP cleavage patterns using the western blot assay. As shown in Figure 2D-2I, the production of sAPPβ and C99 was completely eliminated by treatment with BSI-IV, leading to the accumulation of C83 (Figure 2E, 2F). Treatment with L685,458 caused the accretion of C99 and C83 (Figure 2F). However, treatment with PL402 led to little effect on the level of mature APP (mAPP) and immature APP (imAPP) (Figure 2D) and its cleavage products including sAPPα, sAPPβ, C99 and C83 (Figure 2E, 2F). In addition, TAPI, an α-secretase inhibitor serving as a positive control, significantly reduced the extracellular sAPPα level (Figure 2E) [33]. Data analysis of the western blotting were showed in Supplementary Figure 2A, Figure 2G–2I. Thus, PL402 can reduce Aβ levels without interfering with α-, β-, and γ-secretase enzymatic activity, and also not altering APP metabolism.

### PL402 promotes the expression of MMP3 and MMP9 which are involved in the effect of PL402 on Aβ level modulation

**Figure 3:**
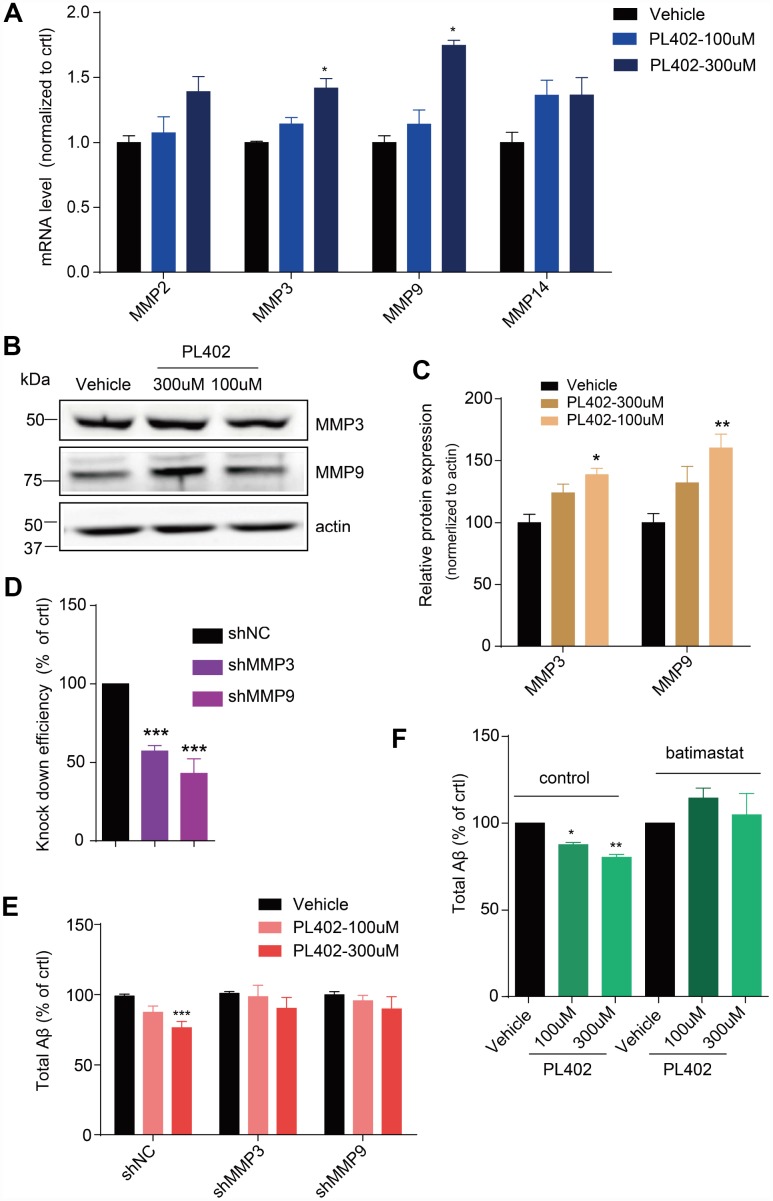
**PL402 promotes the expression of MMP3 and MMP9 which are involved in the effect of PL402 on Aβ level modulation.** (**A**) The mRNA level of Aβ degradation enzymes (MMPs) in SK-N-SH cells treated by vehicle (0.1% DMSO) or PL402 at 100μM and 300μM for 24h. N=4. (**B**–**C**) Representative image of a western blot showing the expression of MMP3 and MMP9 in SK-N-SH cells after treatment with vehicle (0.1% DMSO), or PL402 at 100μM and 300μM for 24h. Actin was used as a loading control (**B**). (**C**) The quantification analysis of (**B**) using ImageJ. N=3. (**D**) The mRNA level of MMP3 and MMP9 in SK-N-SH cells with the infection of scrambled, MMP3 or MMP9 gene-specific shRNA. N=4. (**E**) The levels of total Aβ produced by SK-N-SH cells measured by ELISA after treatment with vehicle (0.1% DMSO) or PL402 at 100μM and 300μM for 24 h in the cells infected with scrambled, MMP3 or MMP9 gene-specific shRNA. N=4. (**F**) The total Aβ level in SK-N-SH cells with presence or absence of the PL402 for 24h after pretreatment with vehicle (0.1% DMSO), or 10μM MMP inhibitor (batimastat) for 1h. N=3. Data are presented as the mean ± SEM, n >3 independent experiments. *p<0.05, **p<0.01, ***p<0.001 compared to the control of each group or the control of the shNC group. One-way ANOVA or two-way ANOVA followed by Bonferroni test.

Aβ level is maintained through a balance of its biosynthesis and degradation and the latter occurs through further enzymolysis by a family of ADEs [16, 34]. To test whether PL402 inhibited the levels of Aβ through influencing the ADEs, we performed RT-qPCR analysis in SK-N-SH cells. As shown in Supplementary Figure 3A, there is no noticeable differences among the mRNA levels of the NEP and IDE after PL402 treatment, but some MMPs, especially the MMP3 and MMP9, significantly increased (Figure 3A). We then examined whether the protein level of MMP3 or MMP9 was increased by PL402 using western blot analysis. The expression of MMP3 and MMP9 was up-regulated obviously by PL402 (Figure 3B, 3C), implying that PL402 decreases the Aβ level through up-regulating the expression of MMP3 and MMP9. To further verify this, we applied shRNAs to knockdown MMP3 and MMP9 in SK-N-SH cells. The knockdown efficiency of the shRNAs was determined by quantitative RT-qPCR. As shown in Figure 3D, 3E, mRNA levels of the two enzymes were respectively reduced after the 72 hours post infection (h.p.i.) (Figure 3D). Interestingly, the knockdown of MMP3 or/and MMP9 blocked the activity of PL402 on Aβ level (Figure 3E, Supplementary Figure 3A), further suggesting that PL402 may function through the two MMPs. Furthermore, we used the effective, broad-spectrum MMP inhibitor, batimastat, to pretreat the SK-N-SH cells for 1 h, and then incubated with the PL402 for 24 h. The culture supernatant was collected to evaluate total Aβ level, and the results showed that the effect of PL402 on Aβ level was abolished by the batimastat pretreatment compared with the control (Figure 3F). All the results suggest that the PL402 may reduce the Aβ level through up-regulating MMP3 and MMP9.

### PL402 ameliorates cognitive deficits and improves memory retention in APP/PS1 mice.

**Figure 4:**
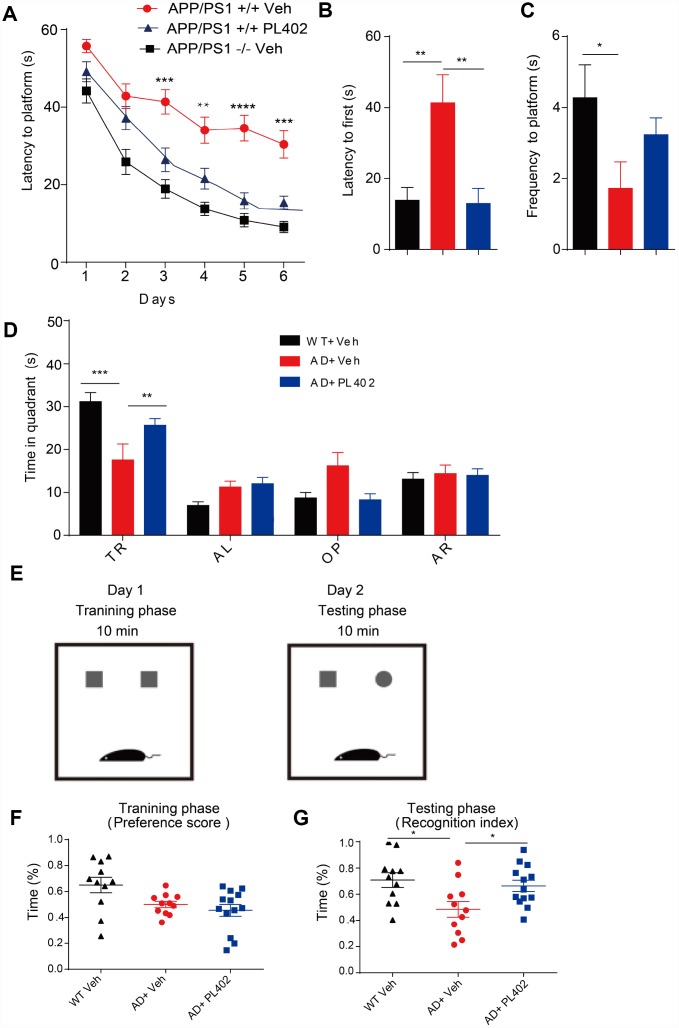
**PL402 ameliorates cognitive deficits and improves memory retention in APP/PS1 mice.** (**A**) Morris water maze (MWM) test of wild type (WT) mice and vehicle- or PL402 treated APP/PS1 mice. (**B**) Latency to platform first of each group mice in day 7 probe trial test. (**C**) Frequency to platform in probe trial for each group of mice in day 7 probe trial test. (**D**) Time spent of each group mice in the target quadrant in day 7 probe trial test. TQ, target quadrant; AR, adjacent right; OP, opposite; AL, adjacent left. Data are presented as mean ± SEM, each group n = 6, ∗p < 0.05, ∗∗p < 0.01, ∗∗∗p < 0.001 analyzed by two-way ANOVA (**A**–**D**) or one-way ANOVA (**B**–**C**) followed by Bonferroni test. (**E**–**G**) Diagram of Novel object recognition (NOR) analysis(E). (**F**–**G**) Preference scores of training phase (**F**) and Recognition Index of testing phase (**G**) during a 10-min testing phase are shown, respectively. Data are presented as mean ± SEM, WT group n = 11, APP/PS1 Vehicle group n = 11, APP/PS1 PL402 group n = 13. ∗p < 0.05, analyzed by one-way ANOVA followed by Bonferroni test.

We then investigated the potential therapeutic effect of PL402 in a mouse model of AD, APP/PS1 transgenic mice. PL402 was orally administered three months in APP/PS1 mice. There were no obvious adverse effects or body weight loss following PL402 treatment (data not shown). First, we examined the spatial learning and memory of treated mice through the Morris water maze (MWM) analysis. We found that compared with the APP/PS1 vehicle mice, WT mice spent less time in locating the platform, indicating that the APP/PS1 vehicle mice exhibited severe cognitive decline in learning. Notably, compared with APP/PS1 vehicle mice, the PL402-treated mice significantly ameliorated learning and memory impairment. And there was no significant difference between PL402-treated mice and WT mice, suggesting that the cognitive function of spatial memory was significantly improved by administration of PL402 (Figure 4A). During the probe trial at day 7, the mice after treatment with PL402 took less time to reach the position of the platform compared with APP/PS1 vehicle mice (Figure 4B), and the PL402-treated mice spent more time in the target quadrant (Figure 4D). Compared with WT mice, APP/PS1 vehicle mice crossed the platform position was less frequently (Figure 4C). However, no significant difference in frequency was observed between PL402-treated mice and APP/PS1 vehicle mice (Figure 4C). These data indicate that PL402 effectively alleviates the spatial learning and memory deficiency of APP/PS1 transgenic mice. To further estimate the effect of PL402 on the learning and memory retention of APP/PS1 transgenic mice, we performed the NOR test (Figure 4E). In the training phase, there was no significant difference among the three groups (Figure 4F). In the testing phase, compared with the WT mice, the APP/PS1 vehicle mice spent less time to contact with the novel object, and meaningfully, the PL402-treated APP/PS1 mice spent much longer time than the APP/PS1 vehicle mice to reach the novel object. Moreover, the recognition index of PL402-treated mice was close to that of WT mice, indicating that PL402-treatment markedly enhanced the learning and memory retention of APP/PS1 mice (Figure 4G).

### PL402 alleviates Aβ burden and promotes Aβ degradation *in vivo.*

**Figure 5:**
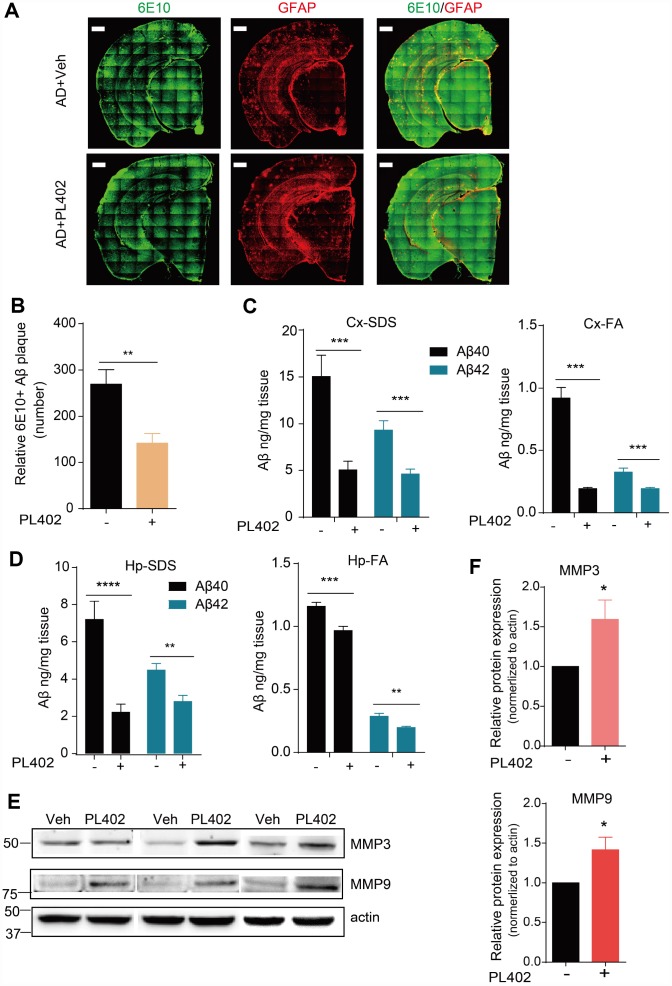
**PL402 alleviates Aβ burden and promotes Aβ degradation *in vivo*.** (**A**, **B**) Representative images (**A**) of Aβ plaques in APP/PS1 mice immunostained with the Aβ antibody 6E10 in coronal mouse brain cryo-sections (n = 5 per group) and the number of Aβ plaques (**B**), were quantified from entire brain sections using Image-Pro Plus 5.1 software (Media Cybernetics), scale bar =500 um. (**C**, **D**) SDS-soluble and FA (formic acid)-soluble Aβ40 and Aβ42 levels in mouse cortex (Cx) and hippocampus (Hp) measured by ELISA. (**E**) The expression of Aβ degradation enzymes (MMP3 and MMP9) in vehicle- or PL402-treated APP/PS1 mice detected by western blot analysis. N=6 mice. (**F**) The quantification of (**E**) using image J. *p<0.05, **p<0.01, ***p< 0.001 and ***p< 0.0001 compared to the control of each group. One-way ANOVA or two-way ANOVA followed by Bonferroni test.

To discover whether PL402 could modulate Aβ deposits *in vivo*, we used the antibody for Aβ named 6E10 to stain for amyloid plaques on the mice brain tissues. Compared with that in the APP/PS1 vehicle mice, the 6E10 positive amyloid plaque numbers were distinctly reduced in the brains of PL402-treated APP/PS1 mice detected by immunofluorescence (Figure 5A, 5B). The result indicates that the PL402 treatment ameliorates the deposition of amyloid plaques. And meanwhile, we performed sandwich ELISA assay to measure Aβ40 and Aβ42 levels in the cortex and hippocampus of vehicle- and PL402-treated APP/PS1 mice. Compared with the APP/PS1 vehicle mice, the PL402-treated mice markedly reduced the SDS (sodium Dodecyl Sulfonate)-soluble and FA (formic acid)- soluble Aβ40 and Aβ42 levels in cortex and hippocampus (Figure 5C, 5D). Moreover, we tried to measure the concentration of truncated Aβ peptides in mouse brain tissues using the mass spectrometry (MS) approach, and the result showed that compared with the APP/PS1 vehicle mice, the PL402-treated mice produced more Aβ degraded fragments (Supplementary Figure 5C). To investigate whether PL402 modulates ADEs level as we observed *in vitro*, we performed western blot analysis of hippocampal tissue from AD mice with or without PL402 treatment, the results showed that the expression of the two enzymes (MMP3 and MMP9) have significant up-regulation compared with the AD mice without PL402 treatment (Figure 5E, 5F). These results suggest that PL402 relieves the Aβ deposits in APP/PS1 mice brain may through promoting Aβ degradation.

## DISCUSSION

In this study, we found that the new rhamnoside derivative named PL402 suppressed Aβ levels in various cell models, however, the expression or activity of β- and γ-secretases and APP metabolism were not influenced by the PL402 treatment, suggesting the effect of rhamnoside derivatives on Aβ irrelevant of its generation. Interestingly, the results show that the levels of ADEs, such as matrix metalloproteinase (MMP) 3/9, were up-regulated by PL402. Furthermore, the effect of PL402 on Aβ level is attenuated by the inhibition of MMP3/9 using either inhibitors to block their activities or shRNAs to knockdown their expressions, indicating that PL402 could reduce Aβ through promoting MMP3/9 level. Meaningfully, PL402 treatment alleviated Aβ pathology and cognitive defects in APP/PS1 mice with the observation of MMP3/9 expression promotion after PL402 treatment. Thus, our data suggest that PL402 could be a promising therapeutic candidate for AD treatment.

In past decades, great efforts have been made to investigate the drugs targeting the pathogenesis of AD [35–38], and a number of candidates aiming at the Aβ clearance have been investigated in animal models or patients with AD [18, 19, 39–41]. The following six mechanisms of Aβ clearance have been reported: enzyme-mediated Aβ degradation, including NEP, IDE, ECE, MMPs, etc.; receptor-mediated Aβ transport; and microglia-dependent phagocytosis; interstitial fluid (ISF) bulk flow and cerebrospinal fluid (CSF) absorption by the circulatory and meningeal lymphatic drainage system [42–44]. Based on accumulating evidence, microglia internalize soluble and fibrillar Aβ *in vivo* and *in vitro* by phagocytosis [19, 34, 45, 46]. In the previous study, Bexarotene increases the removal of soluble Aβ by microglia in an ApoE-dependent manner, and sodium rutin ameliorates AD-like pathology by enhancing microglial Aβ clearance [19, 23]. These evidence suggests that the strategy of targeting Aβ clearance is a promising therapy for AD. In this study, we found that the PL402 could suppress Aβ level in human cell lines (Figure 1C–1I) and AD mice brain (Figure 5A, 5B) through regulating the Aβ degradation by targeting ADEs, especially MMP3 and MMP9 (Figures 3A, 3B and 5E). And the result for mass spectrometry (MS) approach which measure the concentration of truncated Aβ peptides for mouse brain tissues showed that the PL402 treated APP/PS1 mice produced more Aβ degraded fragments than APP/PS1 vehicle mice (Supplementary Figure 4B). These findings will have important implications for the future direction of AD therapeutics based on modulation of MMP bioactivity.

A large body of experimental and clinical evidence has implicated MMPs in tumor invasion, neoangiogenesis, and metastasis, and therefore they represent ideal pharmacologic targets for cancer therapy [1], and the overexpression of MMP plays an important role in the context of tumor invasion and metastasis. Thus, whether the up-regulation of MMP 3/9 by PL402 has some undesired effects may worth further investigation. Some reports suggest that there is an abundance of MMPs in the blood vessel membrane walls in the brain, and the elevation of MMPs levels causes the BBB breakdown which in turn influences Aβ clearance and modulates the accumulation of Aβ in the brain [2]. So, analyzing the expression of MMP3/9 in the cerebral blood vessels and other parts could be also important and requires further verification.

Furthermore, in the recent decays, people start to realize that AD is a complicated brain disorder and single target drug may not effectively treat AD [3, 47–49]. Our laboratory has spent a few years on studying the beneficial effects of natural products on AD treatment and we as well as several other groups found natural products could achieve multi-targets [32, 50, 51]. We recently reported an analogue derived from phenylpropanoids named PL201A, also belonging to rhamnoside derivatives, can improve cognition in transgenic AD mice, promote neurogenesis and protect the mitochondrial functions [28]. Together with the present study showing the activity of the two rhamnoside derivatives on Aβ pathology, we demonstrate that rhamnoside derivatives are strong candidates for AD therapy with multiple function. And in addition to Aβ degradation, we suppose there are other mechanisms contribute to the improved cognitive function in AD mice after PL402 administration which requires further investigation.

## MATERIALS AND METHODS

### Ethics statement

In this study, all animal experiments were performed exactly according to the National Institutes of Health Guide for the Care and Use of Laboratory Animals. The protocols of animal experiments were permitted by the bioethics committee of Shanghai institute of biological sciences, Chinese academy of sciences, with minimizing the pain and discomfort of the experimental animals [32].

### Synthesis of PL402

A sufficient flowchart describing the synthesis of PL402 is provided in Supplementary methods.

### Cell culture

HEK293/APPswe was a cell line stably expressing Swedish mutant form of APP which was transfected into HEK293. SK-N-SH and SH-SY5Y were two human neuroblastoma cell lines and purchased from ATCC. HEK293/APPswe and SK-N-SH were cultured in MEM, and SH-SY5Y in MEM/F12 with 10% (v/v) heat-inactivated fetal bovine serum (FBS) in a humidified incubator with 5% CO2/95% air (v/v) at 37°C. Human iPSC-derived neural stem cells (hNSCs) were maintained as adherent culture in 50% DMEM-F12 and 50% Neurobasal®-A, containing 1x N2 supplement, 1x B27 supplement (Minus Vitamin A), 1x NEAA, 1x Glutamax, 10 ng/ml FGF-Basic (AA10-155) Recombinant Human Protein (bFGF, Gibco), 10 ng/ml LIF Recombinant Human Protein (hlif, Gibco), 3 μM CHIR99021 (Selleckchem), 5μM SB431542 (Selleckchem), and 200 μM L-Ascorbic acid 2-phosphate sesquimagnesium salt hydrate (Sigma). For neurospheres assays, cells were cultured in DMEM-F12 with 1x B27 supplement, 20 ng/mL Recombinant Human Protein (EGF, Gibco), 20 ng/mL bFGF, and 10 ng/mL hlif using low-attachment culture dishes (Corning).

### Compounds and antibodies

TAPI-1, L-685,458 and batimastat were purchased from Selleck. BACE inhibitor IV (BSI IV) was purchased from Calbiochem. Cell Titer-Glo was from Promega. The western blotting assays were achieved with the following antibodies: anti-actin (A2066, Sigma), anti-ADAM10 (Ab1997, Abcam), anti-BACE1 N-terminus (AP7774b, Abgent), anti-APP-CTF (A8717, Sigma), anti-Nicastrin(NCT) (N1660, sigma), anti-PS1 N terminal (Covance), anti-Pen2 (P5622, Sigma) anti-sAPPα (IBL), anti-human sAPPβ-wild type (IBL), anti-MMP3 (A6260, ABclonal), anti-MMP9 (A0289, ABclonal).

### Cell viability measurement

HEK293/APPswe and SK-N-SH cells treated by chemicals were exposed to the Cell Titer-Glo Luminescent Assay (Promega) following the manufacturer’s guidelines.

### ELISA for Aβ *in vitro*

HEK293/APPswe cells, SK-N-SH cells, SH-SY5Y cells, and hNSCs were treated with the indicated chemicals at the various concentrations for 24 h. For the measurement of the Aβ level in these cells, the cultured medium was then collected and exposed to a commercial ELISA kit. The measurement was done according to the manufacturer’s instructions. ELISA kits for human total Aβ, Aβ40 and Aβ42 were attained from ExCell Bio.

### Immunoprecipitation of Aβ

Aβ levels were detected by performing immunopreci-pitation of conditioned media before western blot analysis. Therefore, protein A-beads (Amersham Biosciences) and 4G8 antibody (Covance) were used to immunoprecipitate Aβ. After incubating the supernatant with 4G8 antibody overnight at 4°C, the protein A-beads was added into the mixture and further incubated for 6 h at 4°. Following centrifugation, the beads were rinsed with RIPA lysis buffer (Thermo fisher scientific) for five times and then resuspended with the RIPA lysis buffer. Aβ was then immunoblotted by the 6E10 antibody (BioLegend) in Western blot assay.

### Measurement of BACE1 and γ-secretase activity *in vitro*

We used the ELISA-based secretase activity assays to measure the BACE1 or γ-secretase activity in SK-N-SH cells. The total membrane fractions were extracted from the cultured cells treated with the indicated chemicals. The ELISA-based γ-secretase activity assay were carried out as previously reported [52, 53]. For the ELISA-based BACE1 activity assay, we referred to the previously published literature in lab [32].

### Western blot analysis

Human neuronal cell line SK-N-SH were seeded at a density of 1.2×10^5^ cells/well in culture medium. To detect the expression of C99 and C83, the SK-N-SH cells were seeded at a density of 1×10^6^ cells/well. On the following day, the cells were treated with PL402 for another 24 hours followed by washing twice with PBS. The cells were then lysed with Laemmli sample buffer. The cell lysates were boiled and resolved by SDS/PAGE and then transferred to a nitrocellulose membrane. The interested proteins were recognized by corresponding primary antibodies and the blots were analyzed by the chemiluminescent detection (BioRad) of a peroxidase-conjugated, subtype-specific antibody (1:1000, Abmart). And the quantification for WB was used the image J software and normalized to actin.

### RNA extraction, reverse transcription and quantitative real-time PCR (RT-qPCR)

Total RNA was extracted from SK-N-SH cells with TRI Reagent (T9424, Sigma) according to the manufacturer’s protocols. Reverse transcription (RT) was performed using the random hexamer primers which synthesized by Shanghai Sunny Biotechnology Co. Ltd. and PrimeScript™ RT Master Mix (RR036A, Takara). All the target gene transcriptions were quantified by qPCR and then performed on a Stratagene Mx3000P (Agilent Technologies) with a 2× HotStart SYBR Green qPCR Master Mix (ExCell Bio, Shanghai, China). The primers used for the detection of mRNA levels of human genes are listed in Supplementary Table 1.

### Lentivirus and cell infection

HEK293T cells were seeded in 10-cm dishes at a density of 7 × 10^6^ cells. On the next day, the cells were transfected with shRNA constructs and packaging plasmids. The protocol of this transfection was followed by the calcium phosphate transfection procedure [54]. The cells were further cultured for 48 and 72 h to produce the lentivirus, and the culture medium were then filtrated through 0.45-μm filters. The obtained lentiviruses were further concentrated by ultracentrifugation at 27,000 × g for 2 h, and then the pellets were resuspended in 200 μl of sterile PBS and stored at -80 °C. The flow cytometry (FACS) analysis was applied to determine the titers of packaged lentivirus. For the knock-down experiments, SK-N-SH cells were seeded in 6-cm plates and treated with the concentrated lentiviruses in the presence of Polybrene (Sigma, 8 μg/ml). The cultured medium was refreshed after 24 h incubation. The knock-down efficiency after 72 or 96 h post infection (h.p.i.) was determined used the RT-qPCR. The mentioned protocols referred to the previously reported article [54].

### Animals and drug treatment

The APP/PS1 (APPswe/PS1ΔE9) double-transgenic mice (stock no. 004462) were gained from the Jackson Laboratory expressing a chimeric mouse/human amyloid precursor protein (Mo/HuAPP695swe) and a mutant human Presenilin 1 (PS1ΔE9). Heterozygous mice were kept by crossing with C57BL/6 mice. The controls were matched with the age- and gender-similar wild-type (WT) littermates. The mice were allowed to adapt to the laboratory environment before carrying out the experiments. PL402 (with a purity 85%-95%) was dissolved in vehicle (H_2_O). APP/PS1 mice were oral chronically administered with 200 μL of PL402 (50 mg/kg) or vehicle (H_2_O) per 20 g mouse body weight once a day from 3 to 6 months of age (n = 11-15 mice per group) [50].

### Morris water maze (MWM)

The Morris Water Maze (MWM) analysis was carry out by previously reported [32, 50, 55], and the animals were randomly numbered among genotypes and grouped for the test. The water temperature and the room temperature were constant during the whole experiment. The apparatus was filled with water containing small white plastic particles, and on the four directions of the inner pool wall posted with the cues of four different shapes. Probe trials were conducted on day 4. And on day 8, a single round of probe trial was performed. An automated tracking system (Ethovision XT software) was used to detect the mouse swimming paths and other parameters.

### Novel object recognition (NOR)

The Novel Object Recognition test (NOR) is also widely used to estimate the recognition memory and learning in mice. The detailed protocol with modifications is as previously described [50, 51, 56, 57]. The procedure had two phases: training phase and testing phase. In the training phase, location preference means the time of a mouse exploring one object relative to the time of exploring two objects, and in the testing phase, recognition index means the time of a mouse exploring the novel object relative to the time of exploring two objects.

### Immunostaining and image analysis

After behavioral tests, the mice of each group were anesthetized and transcardially perfused with phosphate-buffered saline (PBS) buffer and then with 4% paraformaldehyde (PFA). The brain cryo-sections (30 μm thick) of every experimental mouse were prepared and immunostained using 6E10 (BioLegend, 803002) for amyloid plaques and GFAP (DAKO) for astrocytes. The all obtained images were captured using a Carl Zeiss Z1 microscope (Zeiss). Quantification was performed using Image-Pro Plus 5.1 software (Media Cybernetics). Five to six sections were analyzed per mouse and all assessments were analyzed.

### ELISA for Aβ *in vivo*

ELISA was performed to detected the Aβ40 and Aβ42 in APP/PS1 mice hippocampus and cortex following the protocols as previously reported [58]. The frozen hippocampal and cortical tissue stored at –80 °C were homogenized in 1 ml 2% SDS (dissolved in PBS), and then centrifuged at 1,20,000 g for 60 min at room temperature. The supernatant was collected as the soluble fraction and quantified with human Aβ ELISA kits (ExCell Bio).

### Statistical analysis

All the experiments were repeated at least three times. Data are representative or mean ± SEM. All data were analyzed by Prism 6.0 (GraphPad Software Inc., San Diego, CA). The concentration-response curve was analyzed by three-parameter nonlinear regression. Unpaired Student’s t-test was a used to compare the two data sets. One-way or Two-way analysis of variance (ANOVA) with Bonferroni test was used where more than two datasets or groups were compared. Statistical significance was accepted at p < 0.05.

## Supplementary Material

Supplementary Materials

Supplementary Figures

Supplementary Table 1
