# Correction for: A novel rhamnoside derivative PL402 up-regulates matrix metalloproteinase 3/9 to promote Aβ degradation and alleviates Alzheimer’s-like pathology

**DOI:** 10.18632/aging.102898

**Published:** 2020-03-04

**Authors:** Tingting Hu, Yue Zhou, Jing Lu, Peng Xia, Yue Chen, Xin Cao, Gang Pei

**Affiliations:** 1 State Key Laboratory of Cell Biology, CAS Center for Excellence in Molecular Cell Science, Shanghai Institute of Biochemistry and Cell Biology, Chinese Academy of Sciences, University of Chinese Academy of Sciences, Shanghai 200031, China; 2 Shanghai EW Medicine Co. Ltd, Shanghai 201203, China; 3 Zhongshan Hospital Institute of Clinical Science, Fudan University, Shanghai 200032, China; 4 Shanghai Key Laboratory of Signaling and Disease Research, Collaborative Innovation Center for Brain Science, School of Life Sciences and Technology, Tongji University, Shanghai 200092, China

**Keywords:** correction

**This article has been corrected:** The authors requested the replacement of panel C of Figure 3 because they reversed the inscriptions on this panel.

This correction does not change the content of the publication. The corrected Figure 3 is provided below.

**Figure 3:**
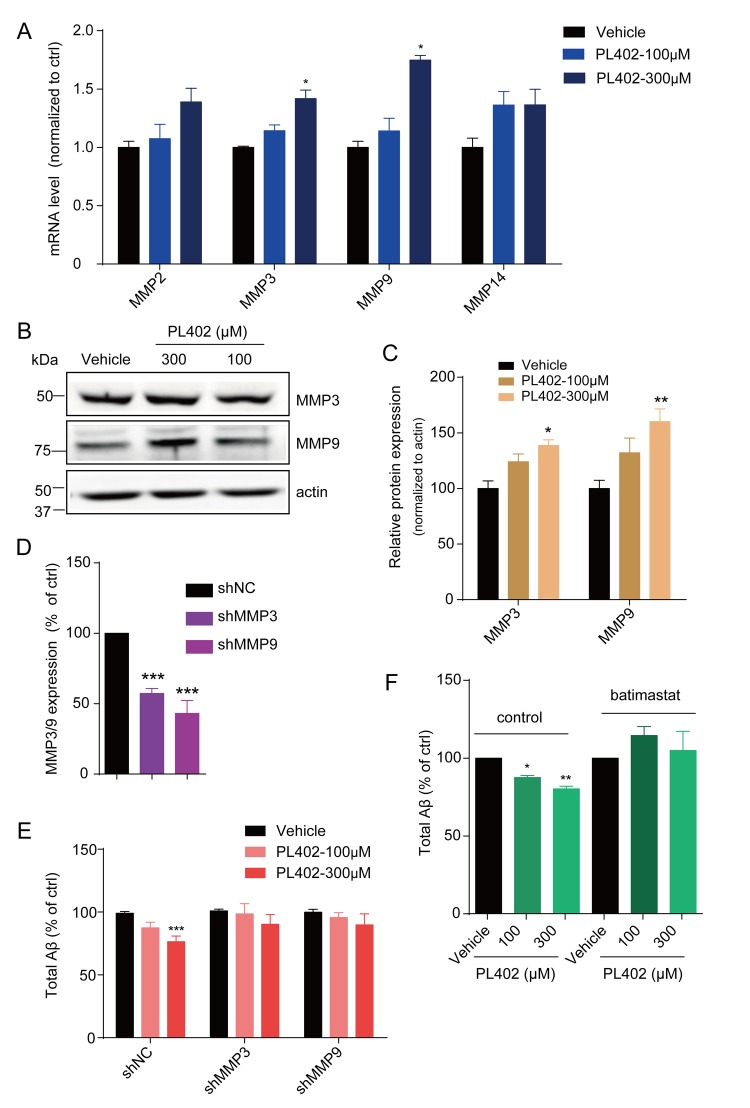
**PL402 promotes the expression of MMP3 and MMP9 which are involved in the effect of PL402 on Aβ level modulation. **(**A**) The mRNA level of Aβ degradation enzymes (MMPs) in SK-N-SH cells treated by vehicle (0.1% DMSO) or PL402 at 100μM and 300μM for 24h. N=4. (**B**–**C**) Representative image of a western blot showing the expression of MMP3 and MMP9 in SK-N-SH cells after treatment with vehicle (0.1% DMSO), or PL402 at 100μM and 300μM for 24h. Actin was used as a loading control (**B**). (**C**) The quantification analysis of (**B**) using ImageJ. N=3. (**D**) The mRNA level of MMP3 and MMP9 in SK-N-SH cells with the infection of scrambled, MMP3 or MMP9 gene-specific shRNA. N=4. (**E**) The levels of total Aβ produced by SK-N-SH cells measured by ELISA after treatment with vehicle (0.1% DMSO) or PL402 at 100μM and 300μM for 24 h in the cells infected with scrambled, MMP3 or MMP9 gene-specific shRNA. N=4. (**F**) The total Aβ level in SK-N-SH cells with presence or absence of the PL402 for 24h after pretreatment with vehicle (0.1% DMSO), or 10μM MMP inhibitor (batimastat) for 1h. N=3. Data are presented as the mean ± SEM, n >3 independent experiments. *p<0.05, **p<0.01, ***p<0.001 compared to the control of each group or the control of the shNC group. One-way ANOVA or two-way ANOVA followed by Bonferroni test.

Original article: Aging. 2020; 12:481–501.  https://doi.org/10.18632/aging.102637

