# Supplementary material for: A novel rhamnoside derivative PL402 up-regulates matrix metalloproteinase 3/9 to promote Aβ degradation and alleviates Alzheimer’s-like pathology
Source: Aging (Albany NY). 2020 Jan 5;12(1):481–501. doi: 10.18632/aging.102637 (PMC6977668; doi:10.18632/aging.102637)
Supplement: Supplementary Materials [file aging-12-102637-s001..pdf]

SUPPLEMENTARY MATERIALS

Synthesis of PL-402

Allyl4-*O*-(2’,3’,4’-tri-*O*-acetyl-α-*L*-rhamnopyranosyl)-α-*L*-rhamnopyranoside (3)

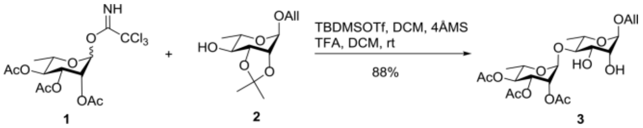

To a solution of compound **2** (7.31 g, 29.95 mmol) in CH_2Cl_2 (75 mL), were added 4 Å MS (15 g) and compound **1** (14.27 g, 32.95 mmol) under N_2 atmosphere. The reaction mixture was stirred at 15 ^oC for 1 h, and then cooled to -5 ^oC. TBDMSOTf (7.3 mL) was added dropwise to the mixture. After stirring for 30min at -5 ^oC, the reaction mixture was warmed up to 10 ^oC, the stirring continued for 2 h. The reaction was quenched by addition of Et_3N. The mixture was filtered. The filtrate was diluted with CH_2Cl_2 and washed with saturated aqueous $NaHCO_3$ solution and brine. The organic layer was dried over Na_2SO_4, filtered, and evaporated *in vacuo* to give the crude disaccharide.

The crude disaccharide (15.14 g, 29.35 mmol) in CH_2Cl_2 (10 mL), was added TFA (13.8 mL). After stirring for 18 h at 20 ^oC, CH_2Cl_2 (15 mL) was added, the reaction mixture was cooled to 0oC and neutralized to pH 8 with saturated $NaHCO_3$ solution, the organic phase was washed with brine, dried over Na_2SO_4, filtered, and evaporated *in vacuo*. The residue was purified by silica gel column chromatography (petroleum ether/ethyl acetat : 10/1-5/1) to give **3** (12.5 g, 88% for two steps) as a yellowish oil. ^1H NMR (400 MHz, $CDCl_3$) δ 5.87 (m, 1 H), 5.33 (m, 1 H), 5.27 (m, 1 H), 5.23 (m, 3 H), 5.08 (m, 1 H), 4.81 (m, 1 H), 4.18 (m, 1 H), 3.96 (m, 3 H), 3.89 (m, 1 H), 3.74 (m, 1 H), 3.52 (m, 1 H), 2.91 (d, *J* = 7.2 Hz, 1 H), 2.65 (d, *J* = 5.9 Hz, 1 H), 2.16 (s, 3 H), 2.11 (s, 3 H), 2.04 (s, 3 H), 1.36 (d, *J* = 6.4 Hz, 3 H), 1.20 (d, *J* = 7.0 Hz, 3 H); ESI-MS *m/z* 477 $(M+1)^+$.

2,3-Di-*O*-acetyl-4-*O*-(2’,3’,4’-tri-*O*-acetyl-α-*L*-rhamnopyranosyl)-*L*-rhamnopyranoside (4)

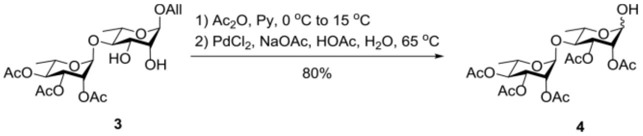

To a solution of **3** (6.84 g, 14.37 mmol) in Py (85 mL), was added acetic anhydride (4.40 g, 43.53 mmol) at 0 ^oC. After stirring for 18 h at 15 ^oC, completion of the reaction was verified by TLC. The reaction mixture was concentrated *in vacuo*. The residue was dissolved in CH_2Cl_2 (100 mL), washed with 0.5 N aqueous HCl solution, saturated aqueous $NaHCO_3$ solution and brine, dried over Na_2SO_4. The solvents were evaporated under reduced pressure to give the protected intermediate.

A mixture of the intermediate (5.04 g, 9.00 mmol), acetic acid (150 mL), sodium acetate (29.50 g, 359.80 mmol), $PdCl_2$ (2.55 g, 14.40 mmol) and water (13 mL), was stirred under an atmosphere of hydrogen (1 atm) at 55 ^oC for 16 h. The mixture was cooled to 20 ^oC, filtered and the filtrate was concentrated under vacuum. The residue was dissolved in CH_2Cl_2 (100 mL), the organic phase was washed with saturated aqueous $NaHCO_3$ solution and brine, dried over Na_2SO_4. The solvents were evaporated under reduced pressure. Purification of the residue by silica gel column chromatography (petroleum ether/ethyl acetate: 10/1-2/1) to give **4** (5.98 g, 80% for two steps) as a yellow foam. ^1H NMR (400 MHz, $CDCl_3$) δ 5.30 (m, 2 H), 5.21 (m, 1 H), 5.10 (m, 3 H), 4.98 (m, 1 H), 4.10 (m, 1 H), 3.98 (m, 1 H), 3.70 (m, 1 H), 3.06 (d, *J* = 4.0 Hz, 1 H), 2.15 (m, 6 H), 2.03 (m, 9 H), 1.36 (d, *J* = 6.4 Hz, 3 H), 1.22 (d, *J* = 7.0 Hz, 3 H); ESI-MS *m/z* 521 $(M+1)^+$.

4’-*O*-tert-Butyldimethylsilyl-ferulic acid (5)

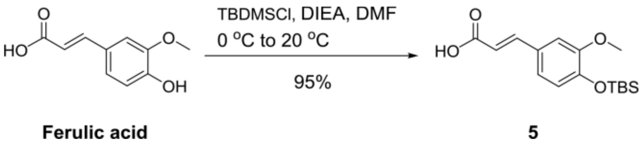

To a solution of ferulic acid (28.00 g, 144.33 mmol) in CH_2Cl_2 (300 mL), were added DIEA (74.47 g, 577.32 mmol), TBDMSCl (43.50 g, 288.66 mmol) at 0 °C, After stirring for 18 h at 20 °C, the reaction mixture was washed with saturated aqueous $NaHCO_3$ solution and brine, dried over Na_2SO_4, filtered, and evaporated *in vacuo*. The residue was purified by silica gel column chromatography (petroleum ether/ethyl acetate : 10/1-2/1) to give **5** (42.23 g, 95%) as a white solid. ^1H NMR (400 MHz, $CDCl_3$) δ 7.72 (d, *J* = 15.8 Hz, 1H), 7.06 (dd, *J* = 8.5, 2.1 Hz, 1 H), 7.05 (m, 1 H), 6.86 (d, *J* = 8.5 Hz, 1 H), 6.31 (d, *J* = 15.8 Hz, 1 H), 3.85 (s, 3 H), 1.00 (s, 9 H), 0.18 (s, 6 H) ; ESI-MS *m/z* 309 $(M+1)^+$.

4’-*O*-tert-Butyldimethylsilyl-ferulate 2,3-di-*O*-acetyl-4-*O*-(2’,3’,4’-tri-*O*-acetyl-α-*L*-rhamnopyranosyl)-α-*L*-rhamnopyranoside (6)

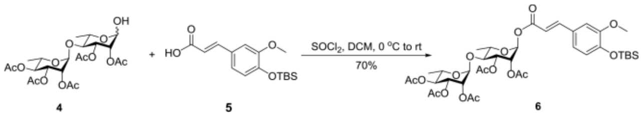

To a solution of **5** (2.09 g, 6.80 mmol) in CH_2Cl_2 (12 mL) at 0 ^oC under N_2 atmosphere, $SOCl_2$ (2.44 g, 20.50 mmol) was added dropwise, the mixture was warmed up to 25 °C, and stirred for 2 h. Completion of the reaction was verified by TLC. The solvents were evaporated under reduced pressure, the residue was dissolved in CH_2Cl_2 (15 mL) and concentrated *in vacuo*. The residue was dissolved in CH_2Cl_2 (12 mL). This solution was added dropwise into a solution of **4** (2.95 g, 5.67 mmol) in CH_2Cl_2 (15 mL) at 0 ^oC under N_2 atmosphere, the mixture was warmed up to 25 °C, the stirring continued for 2 h. The reaction was quenched by addition of water. The organic phase was washed with saturated aqueous $NaHCO_3$ solution and brine, dried over Na_2SO_4, filtered, and evaporated *in vacuo*. The residue was purified by silica gel column chromatography (petroleum ether/ethyl acetate : 10/1-2/1) and **6** (3.85 g, 70%) was obtained as a white foam. ^1H NMR (400 MHz, $CDCl_3$) δ 7.70 (d, J = 16.0 Hz, 1 H), 7.09 (m, 2 H), 6.85 (m, 2 H), 6.36 (d, J = 16.0 Hz, 1 H), 6.09 (m, 2 H), 5.34 (m, 2 H), 5.32 (m, 1 H), 5.11 (m, 3 H), 5.00 (m, 1 H), 3.88 (m, 1 H), 3.83 (s, 3 H), 3.76 (m, 1 H), 2.15 (m, 6 H), 2.05 (m, 9 H), 1.38 (d, J = 6.4 Hz, 3 H), 1.24 (d, J = 6.0 Hz, 3 H), 1.00 (s, 9 H), 0.18 (s, 6 H); ESI-MS *m/z* 811 $(M+1)^+$.

Ferulate 4-*O*-α-*L*-rhamnopyranosyl-α-*L*-rhamnopyranoside (PL-402)

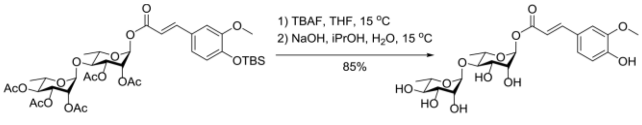

To a solution of **6** (3.21 g, 3.97 mmol) in THF (32 mL), was added TABF (5.94 mL, 1 mol/L). The reaction mixture was stirred at 15 °C for 3 h. Concentration *in vacuo*, the residue was dissolved into ethyl acetate (40 mL) and saturated aqueous $NaHCO_3$ solution (32 mL), the stirring continued for 20 min. The organic phase was washed with water and brine, dried over Na_2SO_4, filtered, and evaporated *in vacuo* to give the phenol intermediate.

To a solution of phenol intermediate (2.63 g, 3.77 mmol) in isopropanol (55 mL), sodium hydroxide (0.79 g, 19.61 mmol) was dissolved in water (55 mL) and added to the solution. After stirring for 1.5 h at 15 °C, the solvents were evaporated under reduced pressure, the residue was dissolved into CH_2Cl_2 (30 mL), washed with saturated aqueous $NaHCO_3$ solution and brine, dried over Na_2SO_4, filtered, and evaporated *in vacuo*. The residue was purified by silica gel column chromatography (petroleum ether/ethyl acetate: 20/1-5/1) to give **PL-402** (1.64 g, 85% for two steps). ^1H NMR (400 MHz, $CDCl_3$) δ 7.57 (d, J = 16.0 Hz, 1 H), 7.14 (d, J = 1.6 Hz, 1 H), 7.10 (d, J = 14.0 Hz, 1 H), 6.73 (d, J = 8.0 Hz, 1 H), 6.29 (d, J = 16.0 Hz, 1 H), 5.94 (d, J = 1.6 Hz, 1 H), 5.13 (d, J = 1.6 Hz, 1 H), 3.88 (m, 1 H), 3.80 (m, 4 H), 3.78 (m, 1 H), 3.63 (m, 2 H), 3.53 (m, 2 H), 3.27 (m, 1 H), 1.21 (d, J = 16.0 Hz, 3 H), 1.16 (d, J = 16.0 Hz, 3 H); ESI-MS *m/z* 487 $(M+1)^+$.
