# Supplementary material for: A novel rhamnoside derivative PL402 up-regulates matrix metalloproteinase 3/9 to promote Aβ degradation and alleviates Alzheimer’s-like pathology
Source: Aging (Albany NY). 2020 Jan 5;12(1):481–501. doi: 10.18632/aging.102637 (PMC6977668; doi:10.18632/aging.102637)
Supplement: Supplementary Figures [file aging-12-102637-s003..pdf]

SUPPLEMENTARY FIGURES

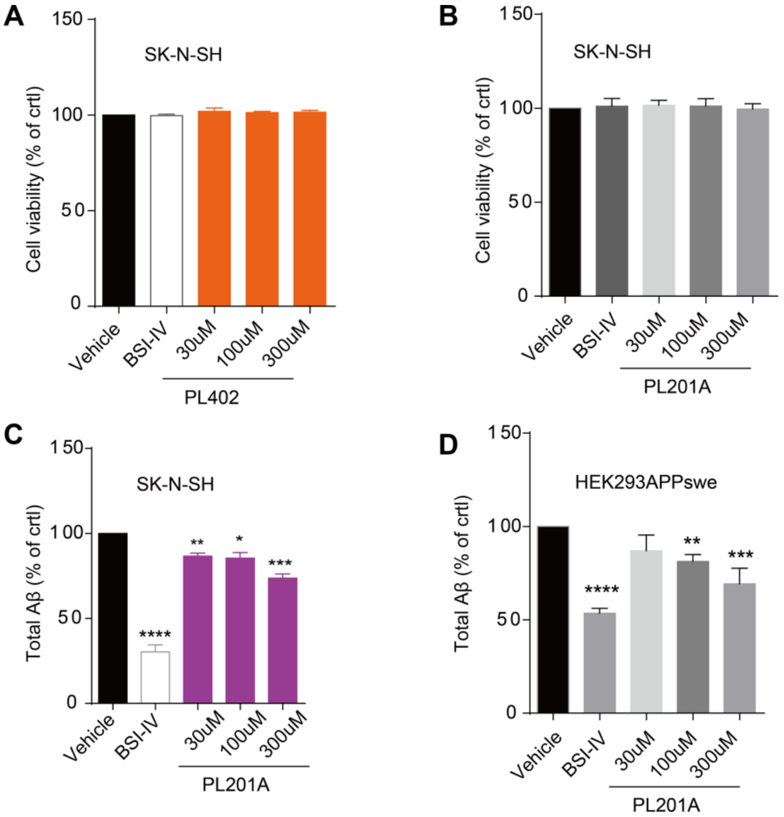

Supplementary Figure 1. The cell viability or Aβ level in response to PL402 or PL201A treatment. (**A**) The cell viability of SK-N-SH cells in response to vehicle (0.1% DMSO), 0.1µM. BACE inhibitor IV (BSI-IV), and the PL402 at 30µM, 100µM or 300µM for 24 hours measured by CellTiter-Glo Assay. (**B**) The cell viability of SK-N-SH cells in response to vehicle (0.1% DMSO), 0.1µM BACE inhibitor IV (BSI-IV), and the PL201A at 30µM, 100µM or 300µM for 24 hours measured by CellTiter-Glo Assay. (**C**) The levels of Aβ produced by SK-N-SH cells in response to vehicle (0.1% DMSO), 0.1µM BSI-IV, and the PL201A at 30µM, 100µM or 300µM for 24 hours. (**D**) The total Aβ level in HEK293/APPswe culture medium treated with vehicle (0.1% DMSO), 0.1µM BSI-IV, or the PL201A at 30µM, 100µM or 300µM for 24h measured by sandwich ELISA. The Data are presented as mean ± SEM, n ≥ 3 independent experiments, *p < 0.05, **p < 0.01, ***p< 0.001 and ****p< 0.001 compared to the control of each group, analyzed by one-way ANOVA followed by Bonferroni test.

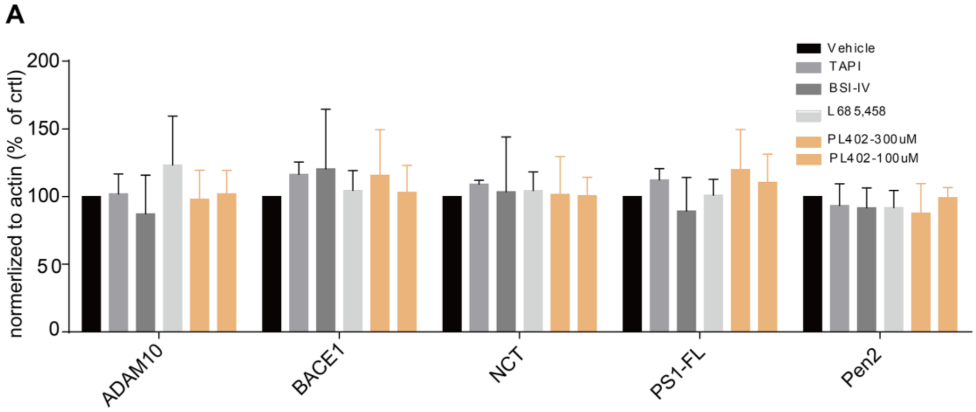

Supplementary Figure 2. The quantification of the α/β/γ-secretase expression for Figure 2C. The statistical analysis for Figure 2C using ImageJ analysis. Data were normalized to the actin.

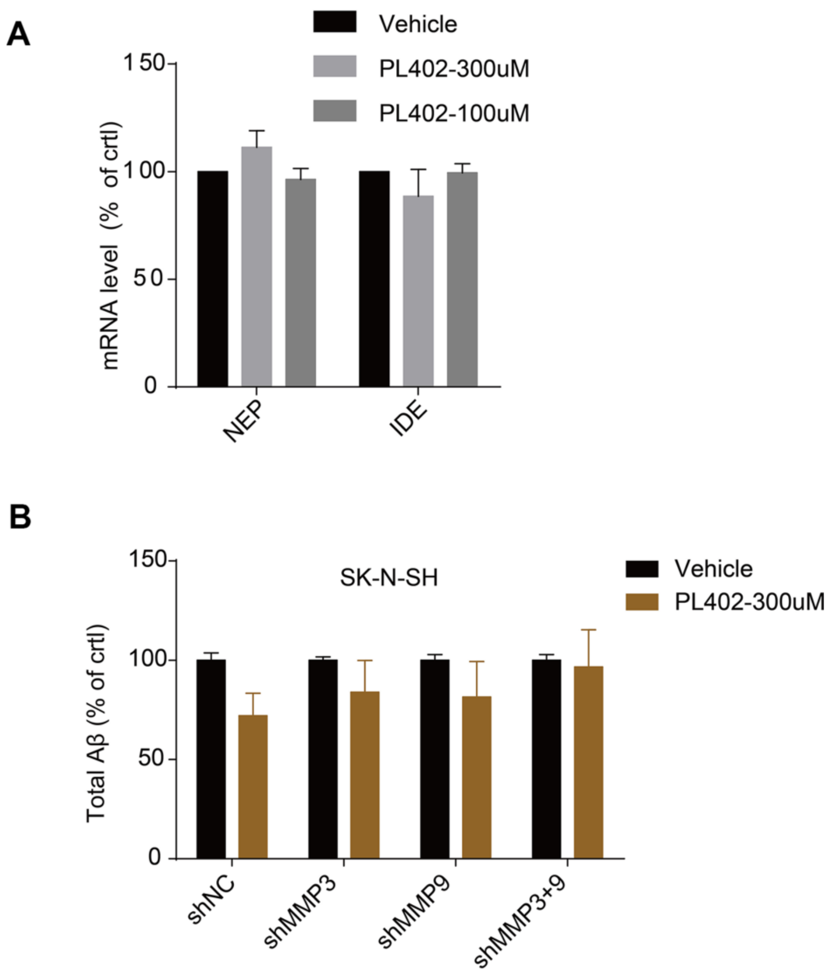

Supplementary Figure 3. The expression of ADEs and the knockdown of MMP3 or/and MMP9 in SK-N-SH cells. (**A**). The mRNA level of Aβ degradation enzymes (NEP and IDE) measured by RT-qPCR in SK-N-SH cells treated with vehicle (0.1% DMSO) or PL402 at 100μM and 300μM for 24h. (**B**). The levels of total Aβ produced by SK-N-SH cells measured by ELISA after treatment with vehicle (0.1% DMSO) or PL402 at 300μM for 24h in the cells which was transfected with the shRNA targeting MMP3 or/and MMP9.

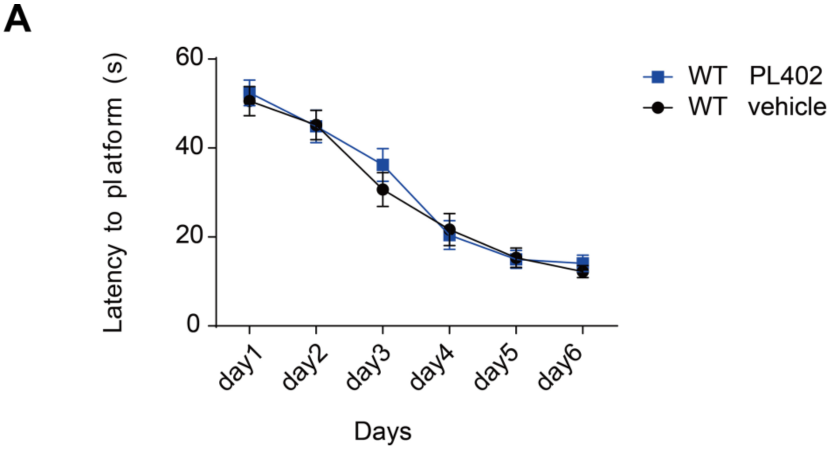

Supplementary Figure 4. PL402 does not affect cognitive function and memory in WT mice. Morris water maze (MWM) test of vehicle- or PL402 treated WT mice (n=8 mice per group).

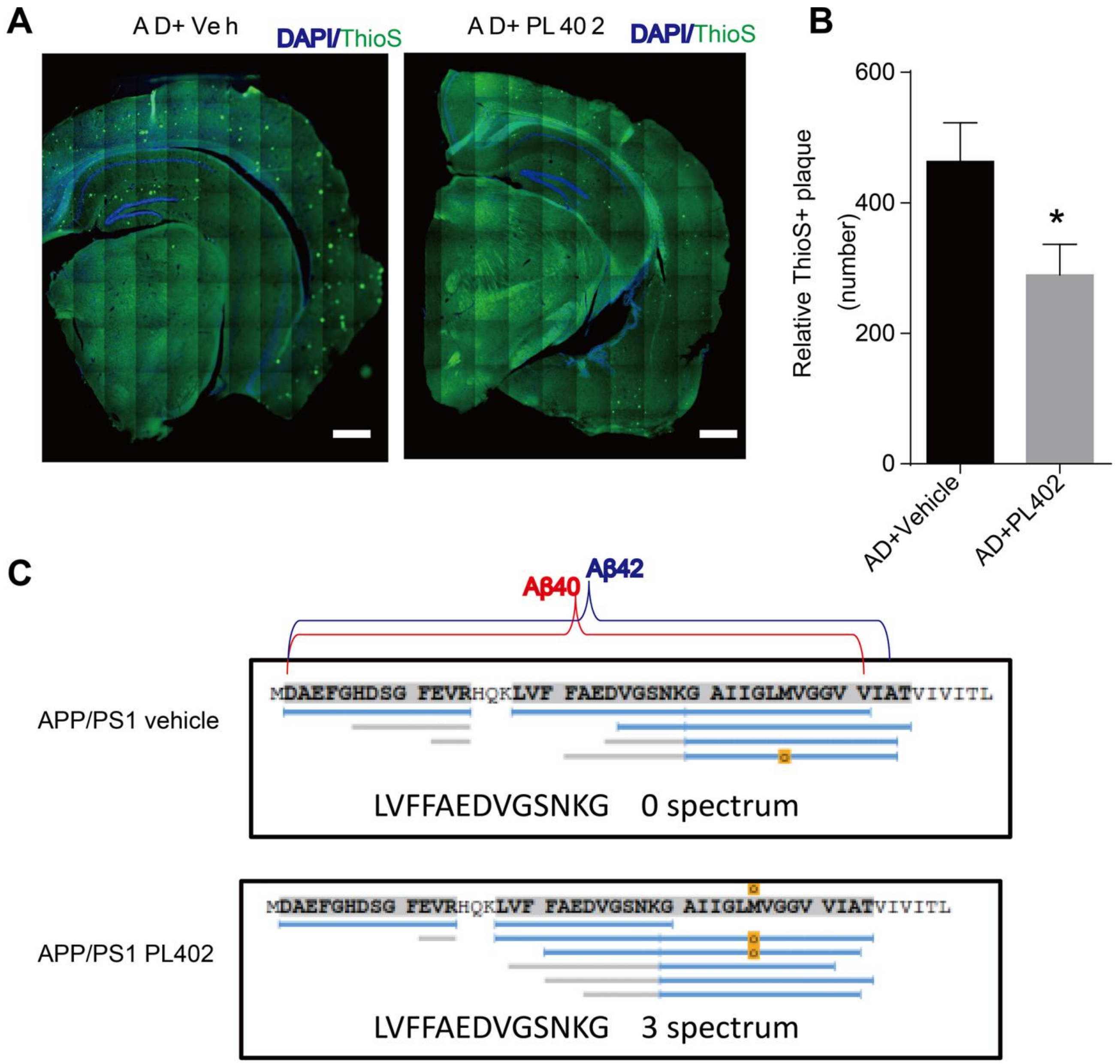

Supplementary Figure 5. PL402 alleviates amyloid plaque burden and promotes Aβ degraded fragments in APP/PS1 mice. (**A**, **B**). Representative images (**A**) of Aβ plaques in APP/PS1 mice stained with the Thioflavin S (ThioS) in coronal mouse brain cryo-sections (n = 5 per group) and the number of Aβ plaques (**B**), were quantified from entire brain sections using Image-Pro Plus 5.1 software (Media Cybernetics), scale bar =500 μm. *p < 0.05 compared to the control group, analyzed by one-way ANOVA followed by Bonferroni test. (**C**). Representatives of truncated Aβ peptides in mouse brain tissues using the mass spectrometry (MS) approach, and the blue lane indicated the various Aβ peptides.
