# Supplementary material for: A novel rhamnoside derivative PL402 up-regulates matrix metalloproteinase 3/9 to promote Aβ degradation and alleviates Alzheimer’s-like pathology
Source: Aging (Albany NY). 2020 Jan 5;12(1):481–501. doi: 10.18632/aging.102637 (PMC6977668; doi:10.18632/aging.102637)
Supplement: Supplementary Table 1 [file aging-12-102637-s002..pdf]

SUPPLEMENTARY TABLE

Supplementary Table 1. Primers used for RT-qPCR

Gene	Direction	Sequence
Human *MMP2*	Forward	TACAGGATCATTGGCTACACACC
	Reverse	GGTCACATCGCTCCAGACT
Human *MMP3*	Forward	CTGGACTCCGACACTCTGGA
	Reverse	CAGGAAAGGTTCTGAAGTGACC
Human *MMP9*	Forward	TGTACCGCTATGGTTACACTCG
	Reverse	GGCAGGGACAGTTGCTTCT
Human*MMP14*	Forward	GGCTACAGCAATATGGCTACC
	Reverse	GATGGCCGCTGAGAGTGAC
Human *NEP*	Forward	AGAAATGCTTTCCGCAAGGCC
	Reverse	AGCCTC CCCACAGCATTTTCC
Human *IDE*	Forward	AGCAGGCTTGAGCTATGATCT
	Reverse	GTTCAGCCCGGAAATTGTTAAGA
ShRNA *MMP3*	Forward	CCGGAGGATACAACAGGGACCAATTCTCG AGAATTGGTCCCTGTTGTATCCTTTTTTG
	Reverse	AATTCAAAAAAGGATACAACAGGGACCAATT CTCGAGAATTGGTCCCTGTTGTATCCT
shRNA *MMP9*	Forward	CCGGGCCGGATACAAACTGGTATTCCTCGA GGAATACCAGTTTGTATCCGGCTTTTTG
	Reverse	AATTCAAAAAGCCGGATACAAACTGGTATT CCTCGAGGAATACCAGTTTGTATCCGGC
